# Passive tobacco exposure may impair symptomatic improvement in patients with chronic angina undergoing enhanced external counterpulsation

**DOI:** 10.1186/1471-2261-8-23

**Published:** 2008-09-17

**Authors:** Stilianos Efstratiadis, Elizabeth D Kennard, Sheryl F Kelsey, Andrew D Michaels, the International EECP Patient Registry-2 Investigators

**Affiliations:** 1 Department of Medicine, Division of Cardiology, University of Utah, Salt Lake City, Utah, USA; 2 Department of Epidemiology, University of Pittsburgh, Pittsburgh, Pennsylvania, USA

## Abstract

**Background:**

The adverse effects of tobacco abuse on cardiovascular outcomes are well-known. However, the impact of passive smoke exposure on angina status and therapeutic response is less well-established. We examined the impact of second-hand smoke (SHS) exposure on symptomatic improvement in patients with chronic ischemic coronary disease undergoing enhanced external counterpulsation (EECP).

**Methods:**

This observational study included 1,026 non-smokers (108 exposed and 918 not-exposed to SHS) from the Second International EECP Patient Registry. We also assessed angina response in 363 current smokers. Patient demographics, symptomatic improvement and quality of life assessment were determined by self-report prior and after EECP treatment.

**Results:**

Non-smoking SHS subjects had a lower prevalence of prior revascularization (85% vs 90%), and had an increased prevalence of stroke (13% vs 7%) and prior smoking (72% vs 61%; all p < 0.05) compared to non-smokers without SHS exposure. Despite comparable degrees of coronary disease, baseline angina class, medical regimens and side effects during EECP, fewer SHS non-smokers completed a full 35-hour treatment course (77% vs 85%, p = 0.020) compared to non-smokers without SHS. Compared to non-smokers without SHS, non-smoking SHS subjects had less angina relief after EECP (angina class decreased ≥ 1 class: 68% vs 79%; p = 0.0082), both higher than that achieved in current smokers (66%). By multivariable logistic regression, SHS exposure was an independent predictor of failure to symptomatic improvement after EECP among non-smokers (OR 1.81, 95% confidence intervals 1.16–2.83).

**Conclusion:**

Non-smokers with SHS exposure had an attenuated improvement in anginal symptoms compared to those without SHS following EECP.

## Background

Epidemiological evidence has unequivocally confirmed that active smoking is a risk factor for cardiovascular disease and the leading cause of preventable death [1,2]. The impact of passive smoking on the cardiovascular system was recognized nearly two decades ago, when evidence of the harmful effects of second-hand smoke (SHS) began to emerge [3,4]. In the past decade, clinical data from the Atherosclerosis Risk in Communities (ARIC) studies demonstrated that both active and passive smoking were associated with accelerated atherosclerosis progression [5]. Steenland et al reported that the risk of death due to cardiovascular disease (CVD) increases by 30% in non-smokers who live together with smokers [6]. Another study suggested that in the United States more than 50,000 deaths annually from ischemic heart disease are associated with SHS [7]. Epidemiological data suggest a non-linear dose-response relationship between the intensity of exposure to SHS and the risk of ischemic heart disease [8,9]. The excess risk of developing CVD amounts to 80% in active smokers at the age of 65, but it may also be as high as 30% in passive smokers [10]. Using measurements of the serum concentration of cotinine, a biomarker of smoke exposure, in a large prospective population study, Whincup et al recently concluded that the risk of coronary heart disease related to passive smoke exposure has probably been underestimated in earlier reports and might, in fact, be very close to the risk reported for active smokers [11].

Enhanced external counterpulsation (EECP) is a non-invasive treatment for chronic, refractory angina that involves the sequential inflation of three sets of lower-extremity cuffs during diastole, leading to increased venous return and cardiac output, systolic unloading, and augmentation of the coronary artery perfusion pressure. EECP has been shown to be effective in treating patients with chronic ischemic coronary artery disease using various measures, including improved functional class [12], reduced anginal symptoms [13], improved quality-of-life indices [14,15], improved stress cardionuclide perfusion [16,17], increased exercise time [18,19], and increased time to ST-segment depression [20]. Treatment with EECP has also been demonstrated to increase nitric oxide levels and decrease malondialdehyde, a marker of lipid peroxidation, as well as to decrease endothelin-1 levels. Moreover, the benefit of EECP has been shown to be sustained at 3- and 5-years after treatment by radionuclide stress testing and quality-of-life measures [21,22].

There have been several emerging predictors of a lower likehood of benefit from EECP treatment including the presence of diabetes mellitus, prior surgical coronary revascularization and heart failure [23]. Moreover, non-smoking patients undergoing EECP treatment had clearly improved symptom reduction compared to current smokers [23]. We examined the impact of second-hand smoke (SHS) exposure on angina status in patients with refractory angina undergoing EECP treatment.

## Methods

The study group consisted of 1,026 non-smoking and 363 current smoking patients treated with EECP for refractory angina enrolled in the Second International EECP Patient Registry (IEPR-II). All patients signed informed written consent prior to entry into the Registry, and the Registry was approved by each center's institutional review board. The primary analyses were performed comparing the non-smokers without SHS exposure (n = 918) and non-smokers with SHS exposure (n = 108). The IEPR-II enrolled consecutive patients treated with EECP for chronic angina in 73 US centers between 2002 and 2004. Briefly, the IEPR-II collected patient demographics, medical history, and coronary artery disease status before and after initiation of EECP therapy. Data were collected prospectively on anginal status according to the Canadian Cardiovascular Society Classification (CCSC), anti-anginal medication use, quality of life and adverse clinical events. The present analysis includes patients who were enrolled from sites reporting ≥ 85% clinical follow-up. Since the IEPR aimed to collect data on as broad a range of patients as possible, the criteria for entry were only that the patient gave informed consent and had at least one hour of EECP treatment for chronic angina. The patients were interviewed by telephone 6 months after the last EECP treatment session, and yearly thereafter in order to record anginal status, quality of life, and adverse cardiac events [24]. Non-smoking is defined as no current tobacco smoking, by self-report. SHS exposure was defined as any exposure to a smoker living in the subject's household.

### Statistical analysis

Data are presented as percentages for categorical variables or as mean values and standard deviations for continuous variables. Percentages and means are for patients with that data item recorded. Data were missing for fewer than 3% of each variable collected. Comparison of continuous variables between groups was analyzed by Wilcoxon rank tests and for categorical variables by chi-square, Fisher's exact test or Mantel-Henzel tests, as appropriate. Multivariable logistic regression analysis was used to determine independent predictors of failure to achieve reduction in angina. All baseline variables associated with the outcome with a p-value of < 0.2 were entered into the preliminary model. A backward selection procedure was used to determine independent, statistically significant predictors. Two-tailed p-values < 0.05 were defined as significant.

## Results

Of the 1,026 non-smoking patients who underwent EECP treatment for angina, 108 (10.5%) reported current SHS exposure. Demographic and clinical characteristics, such as the prevalence of hypertension, diabetes, prior myocardial infarction, heart failure, and anginal class, were similar among the two groups of non-smokers (Table 1). There was a slight trend toward more women in the SHS group (47%) compared to the non-SHS non-smokers (26%; p = 0.74). The SHS subjects had a lower mean age (66 ± 13 vs 68 ± 11 years, p = 0.033), with a lower proportion of patients older than 65 years old (53% vs 63%; p = 0.039). SHS subjects had a higher percentage of previous smoking and prior stroke, but had less reported hyperlipidemia. There was no difference in baseline angina severity between those with and without SHS exposure (Table 2).

**Table 1:** Baseline Demographics and Clinical Characteristics of Non-Smokers Undergoing Enhanced External Counterpulsation

**Variables**	**No SHS Exposure (n = 918)**	**SHS Exposure (n = 108)**	**p-value**
Age (years ± SD)	68.1 ± 10.7	66.1 ± 11.7	0.033
Male (%)	73.6	65.7	0.081
Hypertension (%)	81.4	83.2	0.66
Hyperlipidemia (%)	93.3	87.6	0.035
Diabetes mellitus (%)	43.6	40.7	0.57
Carotid artery disease (%)	24.9	24.3	0.90
Stroke (%)	6.8	13.2	0.018
Prior smoking (%)	61.0	72.4	0.023

Values are expressed as mean ± SD or percentage

**Table 2:** Baseline Coronary Disease Factors and Revascularization Status of Non-Smokers Undergoing Enhanced External Counterpulsation

**Variables**	**No SHS Exposure (n = 918)**	**SHS Exposure (n = 108)**	**p-value**
Interval since CAD diagnosis (yrs)	11.5 ± 8.7	10.9 ± 8.2	0.69
Prior myocardial infarction (%)	69.9	74.8	0.30
Heart failure (%)	24.5	26.4	0.66
Ischemic cardiomyopathy (%)	35.2	41.1	0.23
Left ventricular ejection fraction (%)	47.3 ± 14.4	45.4 ± 14.7	0.25
Prior PCI or CABG (%)	90.4	85.2	0.091
Angina episodes/week	11.8 ± 15.5	13.0 ± 14.2	0.50
Angina class			0.16
I	2.6	1.9	
II	5.1	0.9	
III	68.3	70.4	
IV	24.0	26.9	

Values are expressed as mean ± SD or percentage

More patients without SHS exposure finished the recommended course of at least 35 hours of treatment (85.4% vs. 76.9%, p = 0.020). The rate of major adverse cardiovascular events (myocardial infarction, coronary artery bypass graft surgery [CABG], percutaneous coronary intervention, death) during the course of EECP therapy was 5.6% for the SHS group, compared to 2.8% for the non-SHS group (p = 0.12). However, there was a higher incidence of unstable angina during EECP in the SHS group (8.3%) compared to the non-SHS non-smoker group (2.6%; p = 0.0014). Other adverse events occurring during the EECP treatment period occurred at a similar rate (Table 3).

**Table 3:** Clinical Outcomes after Enhanced External Counterpulsation of Non-Smokers Undergoing Enhanced External Counterpulsation

**Variables**	**No SHS Exposure (n = 918)**	**SHS Exposure (n = 108)**	**p-value**
Death (%)	0.4	1.9	0.12
Myocardial infarction (%)	1.4	1.9	0.67
Unstable angina (%)	2.6	8.3	0.0014
Heart failure exacerbation (%)	1.4	2.8	0.28
Coronary bypass surgery (%)	0.3	0.0	0.55
Percutaneous coronary intervention (%)	1.1	1.9	0.37
MACE (Death/MI/CABG/PCI) (%)	2.8	5.6	0.12
Anginal status (%)			0.044
No angina	16.3	17.9	
Class I	24.2	16.0	
Class II	37.0	31.1	
Class III	17.3	23.6	
Class IV	5.3	11.3	
Angina decreased ≥ 1 class	79.2	67.9	0.0082

Values are expressed as mean ± SD or percentage

**Figure 1:**
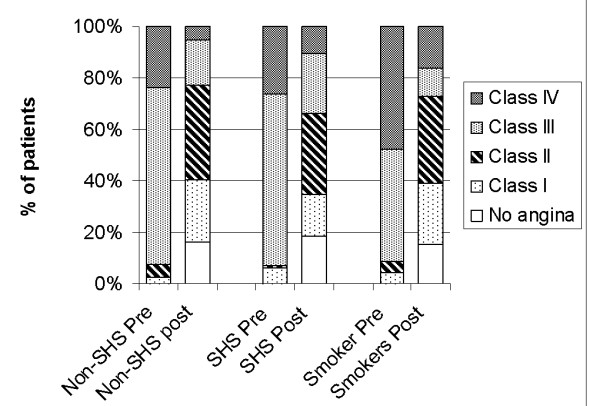
Change in angina class from pre- to post-enhanced external counterpulsation (EECP) treatment for non-smokers without (n = 918) and with (n = 108) second-hand smoke exposure (SHS), and current smokers (n = 363).

After completion of EECP therapy, angina decreased by ≥ 1 class in 68% of the SHS non-smoker group, compared to 79% of the non-SHS non-smoker group (p = 0.0082) both higher than the 66% angina reduction achieved by the current smoking group (Figure 1). Eleven percent of the SHS non-smoker group remained in class IV angina after EECP, compared to only 5% in the non-SHS non-smoker group (p = 0.014). Medical therapy, including beta-blocker, calcium channel blocker, angiotension-converting enzyme inhibitor, angiotensin receptor blocker, antiplatelet, diuretic and hypolipidemic medications, remained similar after EECP in both non-smoking groups.

A multivariable logistic regression model showed the only independent significant predictors of failure to achieve angina reduction were: SHS exposure (odds ratio 1.81, 95% confidence interval 1.16–2.83), age ≤ 55 years (odds ratio 2.10, 95% CI 1.41–3.13), and mild angina (CCSC I/II) pre-EECP (odds ratio 2.70, 95% CI 1.64 – 4.46).

## Discussion

In this large cohort of patients with refractory angina undergoing EECP, SHS exposure was an independent predictor of failure to achieve a reduction in angina class after treatment among non-smokers. Only 68% of those non-smoking subjects with SHS had a reduction in angina class, compared to 79% of the non-smokers without SHS exposure. This low level of angina reduction is comparable to that achieved among current cigarette smokers (66%). Moreover, the SHS group had a higher rate of adverse unstable angina cardiac events during the course of EECP compared to non-smokers without SHS exposure.

Prior clinical studies observed impaired endothelium-dependent vasorelaxation, the earliest sign of endothelial dysfunction, in smokers [25-27]. Passive smokers, defined as those with SHS exposure, exhibited abnormal endothelium-dependent vasodilatation to a similar extent as that seen with current smokers [28]. Sumida et al found that passive smoking changes acetylcholine-induced coronary artery relaxation into vasoconstriction [29]. In another study, coronary flow reserve, although higher in non-smokers than in active smokers under control conditions, was similar in the two groups after a 30-minute exposure to SHS [30]. These findings indicate that the relatively low doses of toxins inhaled by passive smoking are sufficient to elicit a strong acute response. It is noteworthy that the effects of SHS are acute and substantial, nearly as large compared to chronic active smoking (averaging between 80% and 90%) [31].

The agents and pathways responsible for these functional changes of endothelial function have not yet been completely elucidated, but they may be related, at least in part, to the inactivation of nitric oxide (NO) [17]. Animal studies have suggested that passive smoking reduces the activity of endothelial NO synthase [possibly due to the action of carbon monoxide (CO)] [32] and the endothelial arginine content [33]. In accordance with these results, it was shown that L-arginine supplementation prevented endothelial dysfunction induced by SHS in rabbits [32] and reduced the infarct size in SHS-exposed rats [34].

EECP has been shown to increase c-GMP production, which promotes vascular smooth muscle tone and improves arterial function [35]. EECP also progressively increased plasma nitric oxide levels in patients who received 1-hour daily treatments over 6 weeks by 62% compared to baseline improving endothelial function [36]. Therefore, passive smoking could attenuate the vascular effects of EECP by preventing the release of nitric oxide induced by the shear forces generated by EECP.

A limitation of this analysis was the assessment of SHS exposure. By defining SHS exposure as only house-hold exposure, we may have underestimated the proportion of subjects with occupational SHS. However, the vast majority of patients undergoing EECP have severe angina, and are not employed outside the home. Self-reporting of the SHS status may be a limitation. There may be other unmeasured confounding variables, such as obesity, poor diet, and physical inactivity, which may be associated with SHS. Our database was not able to capture these potential confounding variables. The IEPR-2 Registry was designed as a cohort study of patients undergoing EECP and provides a rich data set for exploration. As with many analyses of the Registry data, the SHS hypothesis was not pre-specified; this should be kept in mind in the interpretation of the findings.

## Conclusion

In conclusion, SHS may reduce the anti-anginal effects of EECP among non-smokers. This reduced success rate of EECP in SHS non-smokers is comparable to that seen in current cigarette smokers. These findings are consistent with studies showing that SHS exposure reduces nitric oxide production, which is an important mechanism for the clinical benefit seen with EECP.

## Abbreviations

ARIC: Atherosclerosis Risk in Communities; CABG: Coronary artery bypass graft surgery; CCSC: Canadian Cardiovascular Society Classification; CO: Carbon Monoxide; CVD: Cardiovascular disease; EECP: Enhanced external counterpulsation; IEPR-II: Second International EECP Patient Registry; MACE: Major adverse cardiovascular events; NO: Nitric oxide; SHS: second-hand smoke

## Competing interests

The IEPR-2 is sponsored by Vasomedical, Inc., Westbury, NY. Drs. Michaels, Kennard, and Kelsey have performed consulting for Vasomedical Inc., the manufacturer of EECP. Dr. Michaels has also participated on the speaker's bureau for Vasomedical Inc.

## Authors' contributions

SE drafted the manuscript. EK, SF, and AM conceived of the study, and participated in its design and coordination and helped to draft the manuscript. All authors read and approved the final manuscript.

## Pre-publication history

The pre-publication history for this paper can be accessed here:


